# The protective effect of vagus nerve stimulation against myocardial ischemia/reperfusion injury: pooled review from preclinical studies

**DOI:** 10.3389/fphar.2023.1270787

**Published:** 2023-11-14

**Authors:** Yu-Peng Xu, Xin-Yu Lu, Zheng-Qi Song, Hui Lin, Yi-He Chen

**Affiliations:** ^1^ The First Clinical Medical College, Wenzhou Medical University, Wenzhou, China; ^2^ Department of Respiratory, The Second Affiliated Hospital and Yuying Children’s Hospital of Wenzhou Medical University, Wenzhou, China; ^3^ Department of Cardiology, The First Affiliated Hospital of Wenzhou Medical University, Wenzhou, China

**Keywords:** myocardial I/R injury, vagus nerve stimulation, cardioprotection, meta-analysis, molecular mechanisms

## Abstract

**Aims:** Myocardial ischemia-reperfusion (I/R) injury markedly undermines the protective benefits of revascularization, contributing to ventricular dysfunction and mortality. Due to complex mechanisms, no efficient ways exist to prevent cardiomyocyte reperfusion damage. Vagus nerve stimulation (VNS) appears as a potential therapeutic intervention to alleviate myocardial I/R injury. Hence, this meta-analysis intends to elucidate the potential cellular and molecular mechanisms underpinning the beneficial impact of VNS, along with its prospective clinical implications.

**Methods and Results:** A literature search of MEDLINE, PubMed, Embase, and Cochrane Database yielded 10 articles that satisfied the inclusion criteria. VNS was significantly correlated with a reduced infarct size following myocardial I/R injury [Weighed mean difference (WMD): 25.24, 95% confidence interval (CI): 32.24 to 18.23, *p* < 0.001] when compared to the control group. Despite high heterogeneity (I^2^ = 95.3%, *p* < 0.001), sensitivity and subgroup analyses corroborated the robust efficacy of VNS in limiting infarct expansion. Moreover, meta-regression failed to identify significant influences of pre-specified covariates (i.e., stimulation type or site, VNS duration, condition, and species) on the primary estimates. Notably, VNS considerably impeded ventricular remodeling and cardiac dysfunction, as evidenced by improved left ventricular ejection fraction (LVEF) (WMD: 10.12, 95% CI: 4.28; 15.97, *p* = 0.001) and end-diastolic pressure (EDP) (WMD: 5.79, 95% CI: 9.84; −1.74, *p* = 0.005) during the reperfusion phase.

**Conclusion:** VNS offers a protective role against myocardial I/R injury and emerges as a promising therapeutic strategy for future clinical application.

## Introduction

Myocardial infarction (MI) remains a primary global cause of mortality and disability. Prompt and successful reperfusion of the ischemic myocardium through thrombolytic therapy or primary percutaneous coronary intervention is the most efficacious strategy to salvage ischemic myocardium, mitigate myocardial injury, and enhance clinical outcomes (Hausenloy and Yellon, 2013). However, the process of myocardial reperfusion may trigger cardiomyocyte death and exacerbate cardiac dysfunction. This paradoxical occurrence, known as myocardial ischemia/reperfusion (I/R) injury, curtails the beneficial effects of revascularization strategies (González-Montero et al., 2018; Yang, 2018). While the exact molecular mechanisms of reperfusion-related cardiomyocyte death remain not fully clarified, it thus implicates a pressing need for deep exploration and succedent unmarked novel therapeutic targets (Piper et al., 1998).

Vagus nerve stimulation (VNS) was originally employed for treating refractory epilepsy and depression, leveraging its potential advantages in autonomic neuromodulation (George et al., 2007; González et al., 2019). Subsequent research has increasingly suggested that VNS can also confer protection against heart failure progression, due to the restoration of autonomic balance, baroreceptor sensitivity, and electrical stability (Capilupi et al., 2020; Verrier et al., 2022; Elamin et al., 2023). Recent studies have progressively unveiled the role of VNS in mitigating myocardial I/R injury through the activation of the cholinergic anti-inflammatory pathway, anti-oxidative stress response, or anti-apoptotic response (Chen et al., 2020; Wang et al., 2020; Deng et al., 2022). However, the intricate mechanisms underlying VNS-mediated cardioprotection in experimental studies, along with limited clinical evidence, pose obstacles to its broader application in clinical practice.

Hence, a comprehensive systematic review and meta-analysis are warranted to evaluate the effectiveness of VNS during myocardial I/R injury and provide a deeper understanding of the underlying mechanisms of this therapeutic approach.

## Materials and methods

### Search strategy

We conducted a systematic literature search for animal studies assessing the cardioprotection of VNS in myocardial I/R injury in MEDLINE, PubMed, Embase, and Cochrane Database from the inception to July 2023, with no language restriction. The following search terms were used: “myocardial ischemia/reperfusion injury” OR “myocardial I/R injury” OR “myocardial ischemia-reperfusion injury” AND “vagal nerve stimulation”. Moreover, we searched the references of comments, meeting abstracts, and review articles for additive studies.

### Inclusion and exclusion criteria

Studies were included based on the following criteria: (a) reported the infarct size measured by triphenyl tetrazolium chloride (TTC) and Evan’s blue double staining method, (b) analyzed intervention received VNS treatment merely; comparator intervention received or no treatment, (c) with no cardiovascular-related comorbidity. We excluded studies that did not express infarct size as the percentage of infarct area over the area at risk (AAR) or did not quantify the ischemic area by Evans blue/TTC staining.

### Data extraction

The data were extracted independently by two authors (Yu-Peng Xu and Xin-Yu Lu) from included studies, with discrepancies resolved by consensus. The following details were recorded in Table1: (1) studies’ information, including first author’s name, country, year of publication number of included animals, and duration of I/R injury; (2) animals’ characteristics, including species, gender and anesthetics; (3) the vagal nerve stimulation protocol, including stimulation site, duration, parameters and heart rate reduction; (4) methods for determining the infarct size. The results were expressed in terms of mean and standard deviation to minimize publication bias. The digital ruler software was used to measure the value when some data were only represented by graphs.

**TABLE 1:** Characteristics of included studies, animals and VNS treatment.

Author	Year	Country	Animals	Sample size		I/R duration	Anesthetic agent	Infarct size measurement	VNS protocols			
				Control	VNS				Site of stimulation /Duration	Parameters	HR reduction	Timing of VNS
Bruno et al.Buchholz et al. (2015)	2015	Argentina	Rabbits, NZ, M	10	20	30min/3 h	Pentobarbital	Evans blue/TTC	RVN, 10m, int or con	0.1 m, 10HZ	10%–20%	10min before ischemia
Nederhoff et al.Nederhoff et al. (2019)	2019	Netherland	Mice, C57BL/6, M	19	18	30min/48 h	Fentanyl/Dormicum	Evans blue/TTC	RVN, 30s, con	0.5 m, 10HZ	15%	10min before ischemia
Krekwit et al.Shinlapawittayatorn et al. (2013)	2013	Thailand	Swines	8	16	60min/2 h	Zoletil/Xylazine	Evans blue/TTC	LVN, 3h, int or con	0.5 m, 20HZ	NA	0min after ischemia
Chen et al.Chen et al. (2016)	2016	China	Dogs, mongrel, M	12	9	60min/1 h	Pentobarbital	Evans blue/TTC	LVN, 2h, con	0.1 m, 20HZ	NA	0min after ischemia
Wang et al. (Wang et al., 2014)	2014	China	Rats, SD, M	20	20	30min/2 h	Pentobarbital	Evans blue/TTC	RVN, 30min, con	2.0 m, 10 Hz	10%	15min after ischemia
Zhao et al.Zhao et al. (2013)	2013	China	Rats, SD, M	8	8	60min/2 h	Pentobarbital	Evans blue/TTC	RVN, 3.25h, con	1.0 m, 5HZ	10%	15min before ischemia
Calvillo et al.Calvillo et al. (2011)	2011	Italy	Rats, SD, M	13	6	30min/24 h	Isoflurane	Evans blue/TTC	RVN, 24.7h, con	0.5 m, 8-10HZ	10%	5min before ischemia
Yi et al. (Yi et al., 2016)	2015	China	Rats, SD, M	12	12	30min/4 h	Pentobarbital	Evans blue/TTC	RVN, 30min, con	0.2 m, 10HZ	10%	15min after ischemia
Nuntaphum et al.Nuntaphum et al. (2018)	2018	Thailand	Swines	6	6	60min/2 h	Zoletil/Xylazine	Evans blue/TTC	LVN, 3h, int	0.5 m, 20HZ	NA	0min after ischemia
Krekwit et al.Shinlapawittayatorn et al. (2014)	2014	Thailand	Swines	7	8	60min/2 h	Zoletil/Xylazine	Evans blue/TTC	LVN, 2.5h, int	0.5 m, 20HZ	NA	30min after ischemia

VNS, vagus nerve stimulation; I/R, ischemia/reperfusion; SD, Sprague-Dawley rats; NZ, new zealand rabbit; M, male; RVN, right vagus nerve stimulation; LVN, left vagus nerve stimulation, TTC, triphenyl tetrazolium chloride; HR, heart rate; con, continuous; int, intermittent; NA, none available.

### Quality assessment

Two reviewers independently evaluated and graded the quality of included studies based on published criteria for animal experiments. One point for each of the following: a peer-reviewed publication, random allocation to groups, blinded assessment of outcome, sample size calculation, compliance with animal welfare regulations, and a statement of a potential conflict of interest. Any discrepancies were arbitrated by a third reviewer.

### Statistical analysis

All outcome data were treated as continuous variables in this meta-analysis, presented as the mean and standard deviation. DerSimonian and Laird random effects meta-analysis was used to measure the WMD and the related 95%CIs. Heterogeneity between studies results was evaluated by Cochran’s Q test and quantified by I^2^ statistics test. Begger’s and Egger’s test was used to assess the potential publication bias.

## Results

**FIGURE 1:**
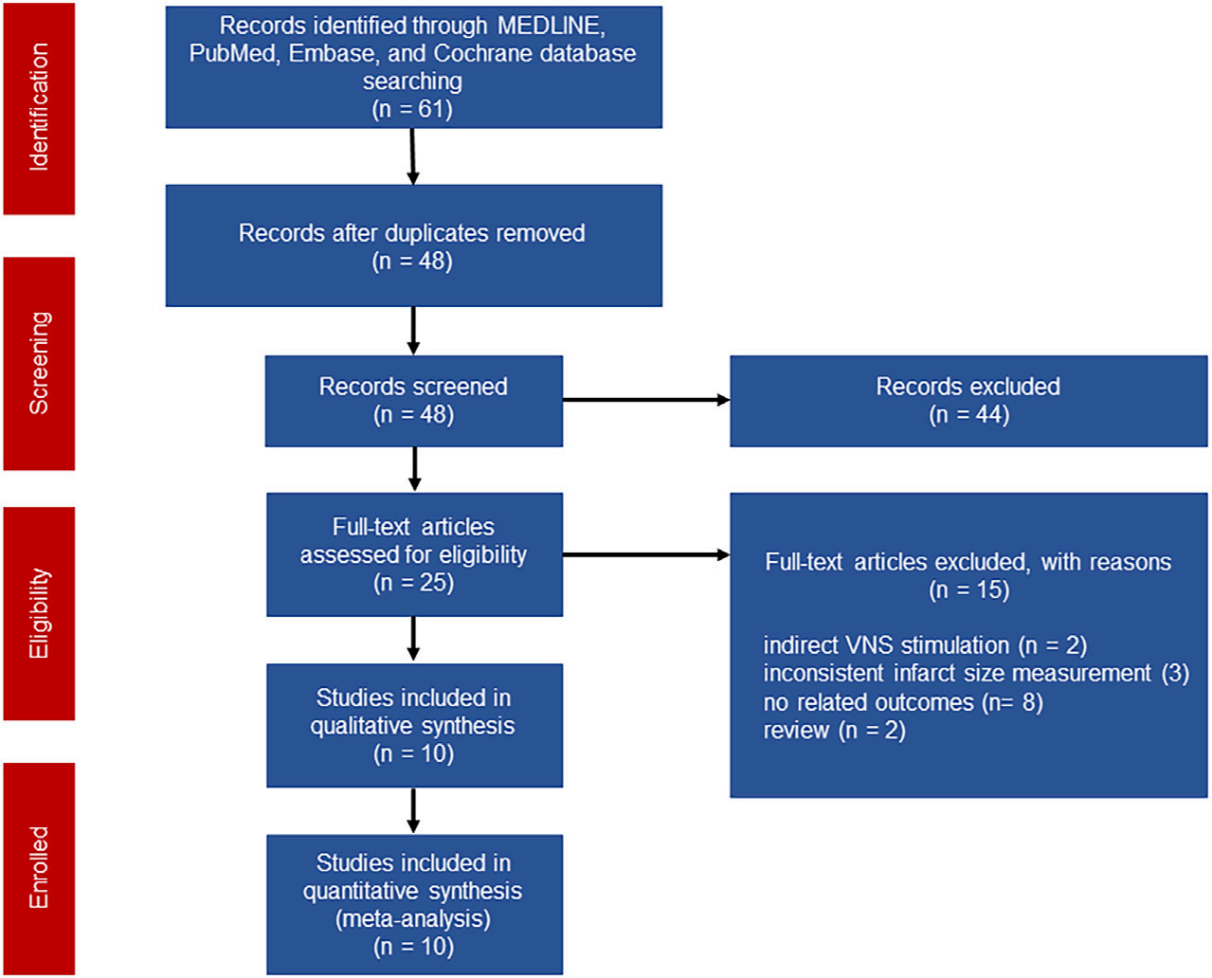
Flow chart of the literature screening.

A total of 61 studies were initially screened and 10 studies comprising 238 animals matched the inclusion criteria for further quantitative analysis (Figure 1). Of these, 123 animals were treated with VNS and 115 animals were treated with control therapy. Cohort characteristics were presented in Table 1. Half of the studies used rodents with the remaining used rabbits, dogs and swine. Continuous, right cervical vagal trunk stimulation was conducted in most of enrolled studies for VNS, the remaining studies performed VNS in left vagal nerve with either continuous or intermittent regimen. The parameters of VNS varied substantially among the studies. The majority of studies reported a 10%–20% heart rate reduction during the procedure to guarantee the biological effect of VNS. Additionally, the potential mechanisms of action of VNS in myocardial I/R injury were detailed in Table 2, predominantly involving anti-inflammatory, oxidative stress, mitochondrial dysfunction anti-apoptosis.

**TABLE 2:** The underlying mechanisms involved in the protective effects of VNS against myocardial I/R injury.

Studies	Year	Proposed mechanisms
Bruno et al	2015	Consistent vagal stimulation: co-activation of the sympathetic nervous system Intermittent vagal stimulation: activation of the Akt/GSK-3β signaling pathway
Nederhoff et al	2019	A less inhibiting effect on inflammatory responsiveness
Krekwit et al	2013	Prevent mitochondrial dysfunction during myocardial I/R
Chen et al	2016	inhibiting oxidative stress and reducing cellular apoptosis
Wang et al	2014	Alleviating inflammatory responsiveness in early phase of myocardial I/R
Zhao et al	2013	Endothelial function and structure protection, anti-inflammatory activity via STAT3 signaling and NF-κB cascade
Calvillo et al	2011	anti-inflammatory and anti-apoptotic activity
Yi et al	2015	Restraining inflammatory cytokines, oxidative stress and apoptosis via IL-17A
Nuntaphum et al	2018	Attenuation of mitochondrial dysfunction, oxidative stress, apoptosis and metabolic abnormalities
Krekwit et al	2014	Protect mitochondrial integrity by mitigating cytochrome c induced apoptosis

VNS, vagus nerve stimulation; I/R, ischemia/reperfusion.

### Infarct size

**FIGURE 2:**
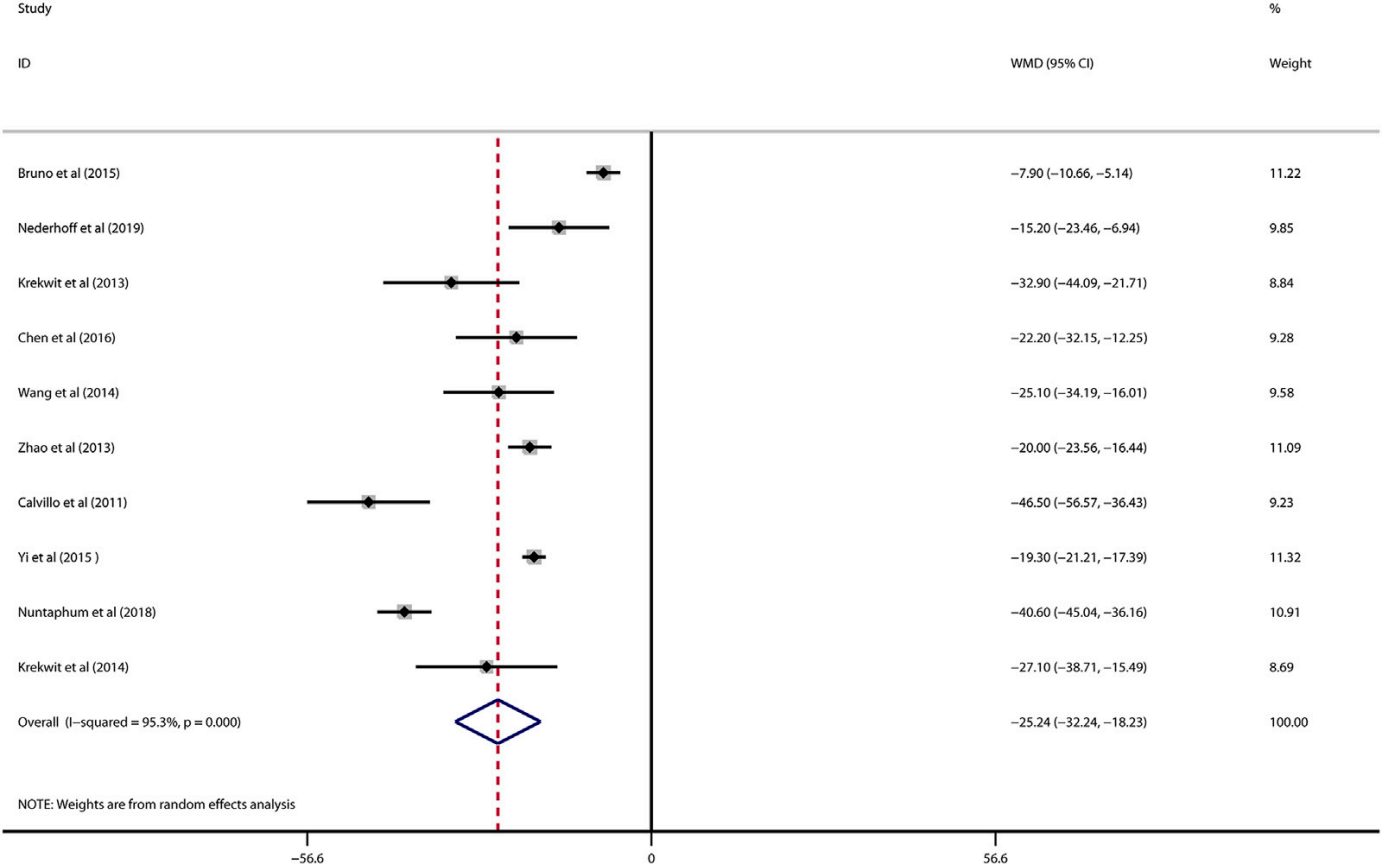
Forest plot of infarct size for VNS treatment against myocardial I/R injury. VNS, vagus nerve stimulation.

Data on infarct size were available in 10 studies. VNS was associated with a dramatic reduction of infarct size assessed by Evans blue/TTC staining post myocardial I/R injury (WMD: 25.24, 95% CI: 32.24 to −18.23, *p* < 0.001, Figure 2), accompanied by high heterogeneity (*I* ^2^ = 95.3%, *p* < 0.001). There was no evidence of publication bias according to Begg’s and Egger’s test. Subsequent sensitivity analysis utilizing the one-study-omit method showed similar findings (Table 3). In addition, stratified analysis according to vagal stimulation site, duration, animal species and state region and myocardial I/R regimen did not influence the efficacy results of infarct size after I/R assaults (Table 4). Further meta-regression also did not reveal any interaction between the pre-specified covaries and VNS-mediated reduction in myocardial I/R damage (Table 5).

**TABLE 3:** Sensitivity analysis for pooled estimates of infarct size by leaving each study out.

Omitted studies	Pooled estimate	95% CI	*p*-Value
Buchholz et al. (2015)	−27.375332	−34.116432; −20.634233	<0.001
Nederhoff et al. (2019)	−26.351851	−33.873882; −18.829815	<0.001
Shinlapawittayatorn et al. (2013)	−24.491714	−31.822363; −17.161068	<0.001
Chen et al. (2016)	−25.56126	−33.027866; −18.094656	<0.001
Wang et al. (2014)	−25.26421	−32.741207; −17.787214	<0.001
Zhao et al. (2013)	−25.974266	−34.273678; −17.674858	<0.001
Calvillo et al. (2011)	−23.037252	−29.975634; −16.098871	<0.001
Yi et al. (2016)	−26.155228	−35.789894; −16.520563	<0.001
Nuntaphum et al. (2018)	−22.940166	−28.790264; −17.090067	<0.001
Shinlapawittayatorn et al. (2014)	−25.065401	−32.455986; −17.674816	<0.001
Combined	−25.235	−32.238; −18.232	<0.001

CI, confidence interval.

**TABLE 4:** Subgroup analysis for pooled estimates of infarct size according to vagal stimulation site, duration, animal species, state region and myocardial I/R regimen.

Pooled estimates	No. of studies	WMD (95% CI)	*p*-Value
VNS type			
Intermittent	4	−32.49 (−44.51; −20.47)	<0.001
Consistent	8	−19.23 (−31.91; −6.55)	0.003
Site of vagus nerve			
RVN	6	−21.24 (−28.15; −14.33)	<0.001
LVN	4	−31.38 (−40.97; −21.79)	<0.001
VNS duration			
>60min	6	−31.49 (−41.67; −21.30)	<0.001
≤60min	4	−16.39 (−24.21; −8.58)	<0.001
Animal			
Small animals	6	−21.24 (−28.15; −14.33)	<0.001
Large animals	4	−31.38 (−40.97; −21.79)	<0.001
Region			
Asian	7	−26.58 (−33.74; −19.42)	<0.001
Europe/America	3	−22.75 (−42.29; 10.14)	0.029
Ischemic duration			
30min	5	−21.83 (−30.66; −13.00)	<0.001
60min	5	−28.62 (−39.31; −17.92)	<0.001
Reperfusion duration			
≥2 h	4	−21.14 (−31.12; −11.15)	<0.001
<2 h	6	−28.05 (−37.16; −18.93)	<0.001
Total	10	−25.24 (−32.24; −18.23)	<0.001

VNS, vagus nerve stimulation; RVN, right vagus nerve stimulation; LVN, light vagus nerve stimulation; WMD, weighed mean difference; CI, confidence interval.

**TABLE 5:** Meta-regression for infarct size.

Covariates	Coefficient	95% CI	*p*-Value
Stimulation type	−7.89749	−20.84117; 5.046191	0.197
Site of vagus nerve	−9.519369	−26.61292; 7.574181	0.235
Duration of VNS	−2.451402	−7.261435; 2.358632	0.274
Species	−3.103634	−9.256419; 3.049152	0.278
Region	−5.766457	−12.56464; 1.031722	0.086
Ischemic duration	−6.530279	−23.95324; 10.89268	0.413
Reperfusion duration	−6.654516	−24.30372; 10.99469	0.410

VNS, vagus nerve stimulation; CI, confidence interval.

Coefficient* indicates the estimates (WMD) of corresponding covariates for infarct size in the context of meta-regression.

### Cardiac function

**FIGURE 3:**
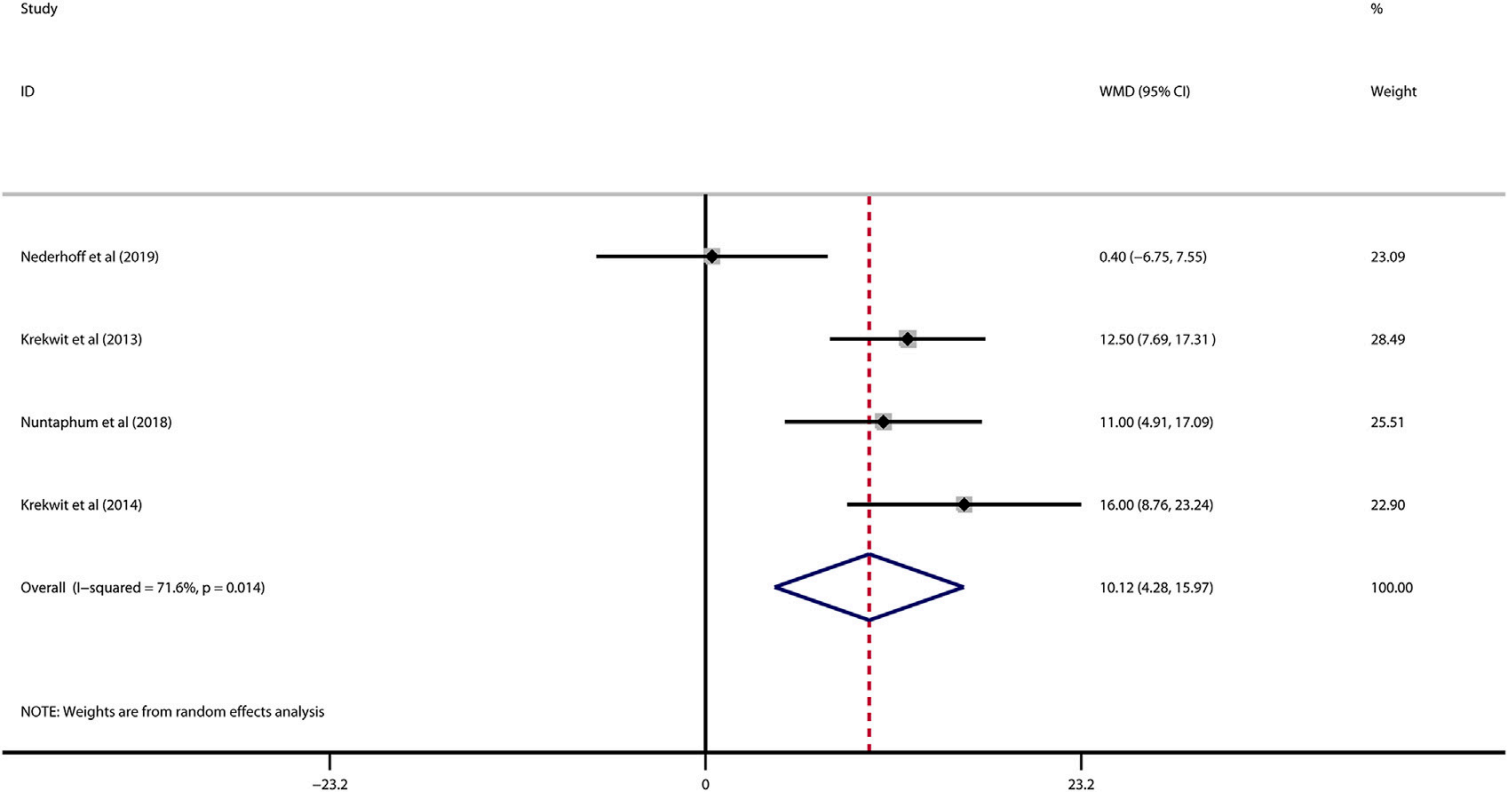
Forest plot of LVEF for VNS treatment in myocardial I/R injury. LVEF: left ventricular eject fraction; VNS, vagus nerve stimulation.

**FIGURE 4:**
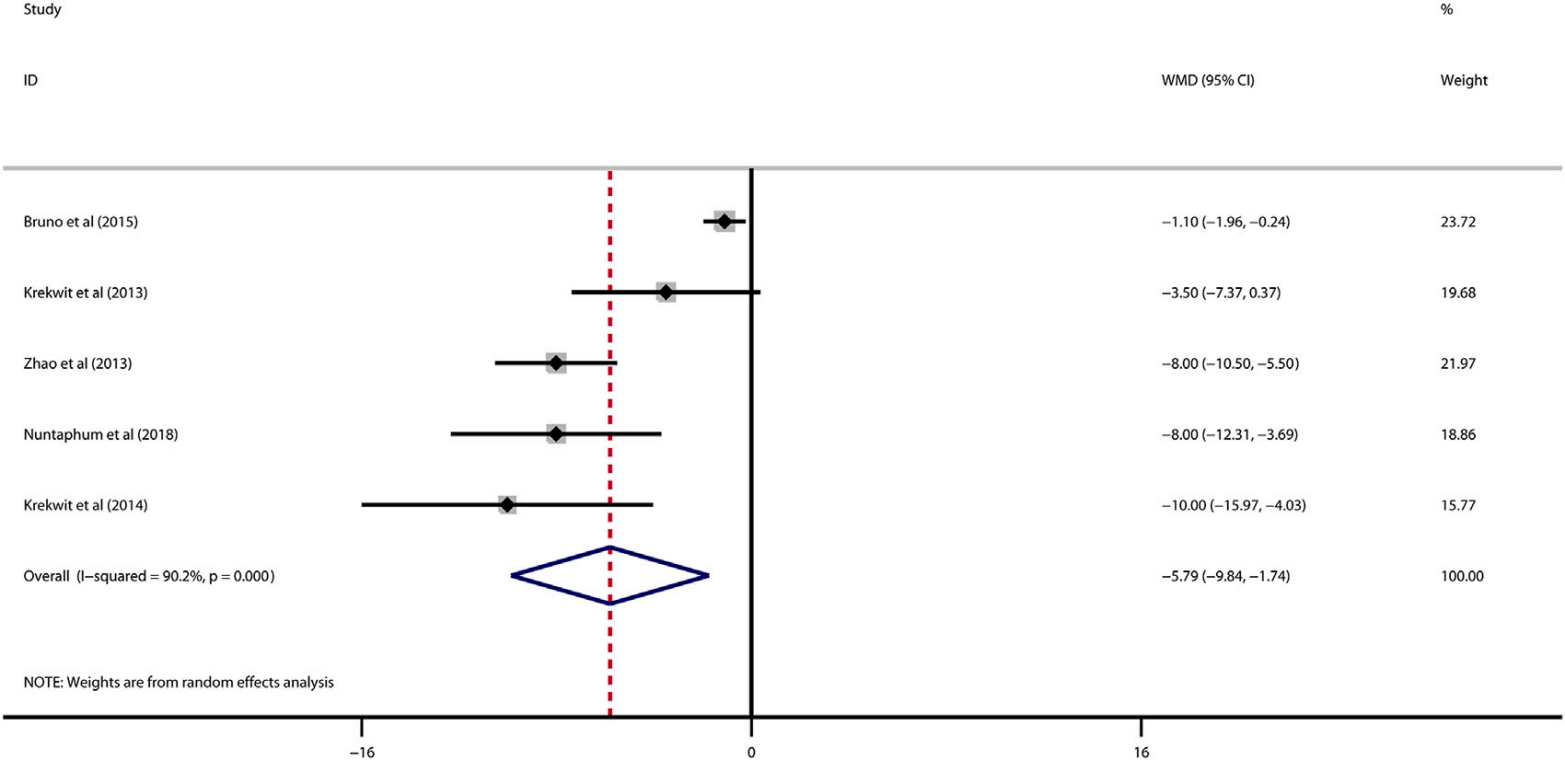
Forest plot of LVEDP for VNS treatment in myocardial I/R injury. LVEDP, left ventricular end-diastolic pressure; VNS, vagus nerve stimulation.

Data on left ventricular ejection fraction (LVEF) was available in 4 studies. VNS was associated with a significantly improved systolic function after myocardial I/R injury (WMD: 10.12, 95% CI: 4.28 to 15.97, *p* < 0.001, Figure 3), with high heterogeneity (*I* ^2^ = 71.6%, *p* < 0.001). Data on left ventricular end-diastolic pressure (LVEDP) were available in 5 studies. In accordance with the results for LVEF, there was also a significantly diminished LVEDP in VNS treated group (WMD: 5.79, 95% CI: 9.84 to −1.74, *p* = 0.005, Figure 4), despite high heterogeneity (*I* ^2^ = 90.2%, *p* < 0.001). One-study-omit sensitivity analysis presented similar results (Table 6). Moreover, there were no signs of any correlation between the pre-specified covaries and both pooled estimates for LVEF and LVDEP, respectively (Table 7).

**TABLE 6:** Sensitivity analysis for left ventricular ejection fraction (LVEF) and left ventricular end-diastolic pressure (LVDEP).

LVEF				LVDEP			
Omitted studies	Pooled estimate	95% CI	*p*-value	Omitted studies	Pooled estimate	95% CI	*p*-value
Nederhoff et al. (2019)	12.795615	9.4486408; 16.14259	<0.001	Buchholz et al. (2015)	−7.1386299	−9.619873; −4.6573863	<0.001
Shinlapawittayatorn et al. (2013)	9.1613674	0.56876612; 17.753969	0.037	Shinlapawittayatorn et al. (2013)	−6.4334135	−11.459242; −1.4075845	0.012
Nuntaphum et al. (2018)	9.7538939	1.3396233; 18.168163	0.023	Zhao et al. (2013)	−5.08149	−9.2801981; −.88278198	0.018
Shinlapawittayatorn et al. (2014)	8.3556633	1.491866; 15.21946	0.017	Nuntaphum et al. (2018)	−5.2803035	−9.7766495; −.78395754	0.021
				Shinlapawittayatorn et al. (2014)	−4.9952269	−9.2649097; −.7255435	0.022
Combined	10.124	4.277; 15.971	0.001	Combined	−5.793	−9.842; −1.744	0.005

LVEF, left ventricular eject fraction; LVEDP, left ventricular end-diastolic pressure; CI, confidence interval.

**TABLE 7:** Meta-regression for left ventricular ejection fraction (LVEF) and left ventricular end-diastolic pressure (LVDEP).

LVEF				LVEDP			
Covariates	Coefficient	95% CI	*p*-value	Covariates	Coefficient	95% CI	*p*-value
Stimulation type	6.042812	−3.079621; 15.16525	0.104	Stimulation type	−3.820668	−7.660276; 0.0189403	0.051
Site of vagus nerve	11.20161	−7.079008; 29.48222	0.119	Site of vagus nerve	−2.525814	−14.33199; 9.280359	0.545
Duration of VNS	11.20161	−7.079008; 29.48222	0.119	Duration of VNS	−6.038574	−12.66625; 0.589101	0.063
Animal	11.20161	−7.079008; 29.48222	0.119	Animal	−3.598583	−8.449862; 1.252697	0.099
Region	11.20161	−7.079008; 29.48222	0.119	Region	−3.598583	−8.449862; 1.252697	0.099
Ischemic duration	11.20161	−7.079008; 29.48222	0.119	Ischemic duration	−6.038574	−12.66625; 0.589101	0.063
Reperfusion duration	11.20161	−7.079008; 29.48222	0.119	Reperfusion duration	−6.038574	−12.66625; 0.589101	0.063

LVEF, left ventricular eject fraction; LVEDP, left ventricular end-diastolic pressure; VNS, vagus nerve stimulation; CI, confidence interval.

Coefficient* indicates the estimates (WMD) of corresponding covariates for LVEF, or LVDEP, in the context of meta-regression.

## Discussion

As far as we are aware, this is the first meta-analysis ever conducted to demonstrate that VNS is beneficial in protecting the myocardium from ischemia-reperfusion (I/R) injury. By incorporating data from 10 distinct studies, our research evaluated the efficacy of VNS in preclinical studies. These findings indicated that VNS could significantly reduce infarct size during myocardial I/R injury and also improve heart function by reducing LVEDP and increasing LVEF. Intriguingly, these benefits were observed to be independent of the type and site of VNS or the animal size.

Myocardial I/R injury remains a significant clinical challenge despite advancements in reperfusion therapies such as thrombolysis and PCI(Férez Santander et al., 2004). This is primarily because of the intricated pathophysiologic process underlying reperfusion injury, including oxidative stress, calcium overload, inflammation, mitochondrial dysfunction, and cell apoptosis (Davidson et al., 2019). During the reperfusion, excessive reactive oxygen species (ROS) production due to the abrupt increase in oxygen supply and corresponding antioxidant enzyme insufficiency are the critical factor of cardiomyocyte death (Férez Santander et al., 2004; Hausenloy and Yellon, 2013). Meanwhile, previous studies reported that mitochondria are the main source of ROS and mitochondrial damage affects post-injury cardiac function by dysregulated ROS modulation. In a vicious cycle, ROS can also impair the mitochondrial respiratory chain and promote mitochondrial membrane depolarization, leading to impaired ATP production and further exacerbating cell death (Murphy and Steenbergen, 2008; Ong and Hausenloy, 2010; Ong and Gustafsson, 2012). Moreover, non-coding RNA, including mi-RNA and Lnc-RNA have increasingly emerged as key regulators in various cellular processes such as apoptosis, inflammation, fibrosis, and angiogenesis and have implications for myocardial ischemia-reperfusion (I/R) injury (Ong et al., 2018). Unfortunately, there are currently limited therapeutic options available to prevent heart damage from reperfusion injury. Several pharmacological interventions have been tried to attenuate myocardial I/R injury by targeting the abovementioned cellular and molecular mechanisms. Vitamins C and E, being well-established antioxidant, have been shown to reduce cardiomyocyte death by inhibiting ROS release during the reperfusion injury (Rodrigo et al., 2014). In addition to the anti-inflammatory drugs, calcium channel blockers or cyclosporine also showed similar cardioprotective effects in retarding infarct area extension and subsequent deterioration of systolic function in a preclinical setting (Boden et al., 2000; Piot et al., 2008; Trelle et al., 2011). However, none of them showed the theoretical potential in clinical translation due to the huge gap between compelling experimental evidence and scant clinical data.

The vagus nerves, originating from the medulla oblongata, are the longest cranial nerve and is involved in the regulation of various physiological systems (Berthoud and Neuhuber, 2000). VNS is first identified as a therapeutic approach for Inflammatory disease by activating the cholinergic anti-inflammatory pathway (Bonaz et al., 2016). On the contrary, vagal denervation consistently released the lymphocyte from thymus to spleen and lymph nodes, which indicated the role of vagus nerves in controlling inflammatory status (Antonica et al., 1994; Antonica et al., 1996). Recently, clinical trials and preclinical trials have demonstrated the beneficial effect of VNS in reducing arrhythmias and hospitalizations, improving cardiac contractility and quality of life for patients with heart failure or AF, which suggests a crucial role of VNS in the treatment of heart disease (Li et al., 2004; Zhang et al., 2009; Zannad et al., 2015; Gold et al., 2016). In terms of the physiological properties of VNS, it was also utilized as a promising method for alleviating myocardial reperfusion injury. As expected, VNS modulates inflammatory cytokines and simultaneously inhibits ROS by activation of AMPK cascades (Kong et al., 2012). Additionally, experimental research indicated that VNS preserved the integrity and function of mitochondria by regulating mitochondrial dynamics, biogenesis, and mitophagy, which turns into cardioprotection against myocardial I/R injury (Nuntaphum et al., 2018). Meanwhile, VNS suppresses the sympathetic nerve sprouting and blocks the inflammatory process, which attenuating ventricular remodeling and decreases the incidence of ventricular arrhythmias after reperfusion injury on mechanism, Jak2/STAT3, NF-κB, Akt/GSK-3β signaling pathway, which are responsible for VNS induced preventive effects on myocardium during reperfusion injury (Buchholz et al., 2015; Zhao et al., 2021). Yoshihiko et al. reveal a PI3K/Akt pathway for HIF-1α induction by vagal stimulation, which minimizes cardiomyocyte apoptosis under hypoxia and normoxia (Kakinuma et al., 2005). Intriguingly, *in vitro* studies also have demonstrated that VNS could impede FoxO3A phosphorylation through P13K/AKT signaling activation, thus optimizing the sequelae of infarct myocardium (Luo et al., 2020). Collectively, preclinical evidence confirms the potential ability of VNS in facilitating heart recovery from I/R damage, and raise the possibility that it may have a role in improving the prognostic endpoints of myocardial infarction patients receiving timely revascularization. In accordance with the animal experimental results, Yu et al. have reported that tragus stimulation significantly reduces the inducibility of reperfusion-induced ventricular tachycardia and the levels of myocardial injury biomarkers, improves systolic function in patients with STEMI undergoing PCI(Yu et al., 2017).It also indicates that suppressed inflammatory response, evidenced by lower IL-6, IL-1β, high-mobility group-box 1 protein 1, and TNF-α, contributes to the favorable effects of tragus stimulation. However, there remains a great challenge to translate the cardioprotective effects of VNS into myocardial infarction patients, and it therefore is still a pressing need for well-designed randomized control trials to further confirm the role of VNS in the setting of myocardial I/R injury, and contemporaneously deeply elucidate the underlying mechanisms.

### Limitations

First, there is no standard protocol for myocardial I/R regimen (i.e., different ischemic or reperfusion duration) or VNS treatment (i.e., different parameters, stimulation site, and type), while subgroup analysis shows remarkable consistent outcomes among the studies. Second, the pooled results from this meta-analysis are based on animals without comorbidities which may impede extrapolating these findings to complicated clinical situations. Third, despite significant heterogeneity that may affect the interpretation of the results, sensitivity analysis and subgroup analyses with robust data substantially support the benefits and reliability of VNS in reducing infarct size and improving cardiac function after reperfusion injury. Meanwhile, the prespecified covariates have no impact on pooled results of both infarct size and LVEF by meta-regression. Finally, the majority of outcomes of included studies concentrate on infarct area and LVEF, rather than mortality or other cardiac functional indicators (e.g., 6-min walking or cardiopulmonary exercise testing), which may more precisely reflect the prognosis and symptoms in clinical practice.

## Conclusion

In summary, VNS is a promising therapeutic strategy for preventing lethal myocardial reperfusion injury according to the significant advantages in limiting infarct size and cardiac function from basic studies. It thus provides the theoretical feasibility and reliability to extend the utilization of VNS in ST elevation myocardial infarction patients with revascularization, and implicates the future prospects of clinical application.

## Data Availability

The original contributions presented in the study are included in the article/supplementary material, further inquiries can be directed to the corresponding author.
